# Six months in: pandemic crime trends in England and Wales

**DOI:** 10.1186/s40163-021-00142-z

**Published:** 2021-03-04

**Authors:** Samuel Langton, Anthony Dixon, Graham Farrell

**Affiliations:** School of Law, University of Leeds, Leeds, UK

**Keywords:** COVID-19, Time series, ARIMA, Routine activities, Opportunity theory, Pandemic, Mobility

## Abstract

Governments around the world have enforced strict guidelines on social interaction and mobility to control the spread of the COVID-19 virus. Evidence has begun to emerge which suggests that such dramatic changes in people’s routine activities have yielded similarly dramatic changes in criminal behavior. This study represents the first ‘look back’ on six months of the nationwide lockdown in England and Wales. Using open police-recorded crime trends, we provide a comparison between expected and observed crime rates for fourteen different offence categories between March and August, 2020. We find that most crime types experienced sharp, short-term declines during the first full month of lockdown. This was followed by a gradual resurgence as restrictions were relaxed. Major exceptions include anti-social behavior and drug crimes. Findings shed light on the opportunity structures for crime and the nuances of using police records to study crime during the pandemic.

**Supplementary Information:**

The online version contains supplementary material available at 10.1186/s40163-021-00142-z.

## Introduction

The ongoing COVID-19 pandemic has had a profound impact on societies around the world. Physical health, mortality rates, healthcare systems, economic performance, mental well-being, social interactions and mobility have experienced unprecedented change in response to both the virus itself and attempts to control its spread. Evidence has begun to emerge which demonstrates the stark effects of nationwide lockdowns and ‘stay at home’ messages on crime (e.g. Ashby 2020b; Felson et al. 2020; Halford et al. 2020). Here, we take an initial ‘look back’ on crime trends in England and Wales during the first six months of the nationwide lockdown. Findings hold particular significance for the study of opportunity theories and crime, and shed light on the merits and shortcomings of using police-recorded crime data to examine the impact of lockdown measures on criminal behavior.

Curbs on citizens’ mobility and social interactions have been widely deployed by local and national governments to stem the spread of COVID-19. The degree to which countries have mandated and enforced these guidelines has varied, but in the United Kingdom, as in many European countries, the government adopted a legally-enforced “stay at home” ruling for citizens in March. There were exceptions to these restrictions. For instance, essential commercial outlets such as supermarkets could remain open, and ‘key workers’ in professions such as social and health care could continue to work. Generally speaking, countries adopting stay at home policies witnessed a change in citizen mobility and routine activities in a manner which was both instantaneous and unprecedented. These alterations in daily life, globally detectable and recorded using measures of seismic activity, have been described as the ‘great quiet period’ in human mobility (Lecocq et al. 2020).

The pervasiveness and speed of these interventions into citizens’ lifestyles and daily activities represents a unique opportunity to study criminal behavior in an experimental setting (Eisner and Nivette 2020; Stickle and Felson 2020). The global nature of lockdowns, and the variance in timing and severity between countries (and even within countries), make this a particularly fortuitous moment in criminological inquiry. Specifically, the lifestyle (Hindelang et al. 1978) and routine activities (Cohen and Felson 1979) approaches offer useful perspectives from which to understand the impact. Drastic changes to lifestyles, here manifest in terms of human mobility, are likely to yield similarly drastic changes in the scale and nature of interaction between potential targets, motivated offenders and capable guardians (Farrell and Tilley 2020). For instance, stay at home measures boost daytime guardians in residential areas (‘eyes on the street’), potentially *reducing* opportunities for burglary. By the same measure, crimes such as child abuse or intimate partner violence may *increase* as a result of victims and offenders spending more time together in a domestic setting. The fact that it is primarily the rate of interaction of potential targets, offenders and guardians that has changed prompted Halford et al. (2020) to propose a mobility theory of crime during the pandemic.

This paper investigates the impact of the COVID-19 pandemic on crime and anti-social behavior using six months of open police-recorded data in England and Wales. Using historical monthly trends as a baseline for comparison, we quantify the scale and character of change across fourteen different offence types during the nationwide lockdown. Findings are evaluated for their consistency with theoretical expectations and discussed with a critical eye to using police-recorded crime data and a natural experiment research design. The study evolved from work developed for two *Briefings* series that were established early in the pandemic for policy-makers and practitioners. In the *JDI Special Papers* series we outlined an initial typology of pandemic crime effects (Farrell 2020) and models of post-lockdown crime outcomes (Farrell and Birks 2020), and in the *Statistical Bulletin on Crime and COVID-19* we explored emerging issues in crime trends and its distribution nationally and locally (e.g. Dixon et al. 2020a, b, c; Dixon and Farrell 2020a; Langton 2020; see also Farrell et al. 2020).^1^

## Literature review

In recent months, studies have emerged internationally which have helped establish the extent to which crime and calls to police during lockdowns have deviated from expected trends (see Table 1). These contributions have largely featured case study sites in the United States, including San Francisco and Oakland (Shayegh and Malpede 2020), Los Angeles (Campedelli et al. 2020; Mohler et al. 2020), Detroit (Felson et al. 2020), Indianapolis (Mohler et al. 2020), Dallas (Piquero et al. 2020) and Chicago (Bullinger et al. 2020), some examining multiple cities (Ashby 2020a, b) and nationwide (Hawdon and Dearden 2020). Studies have also been conducted in the United Kingdom (Buil-Gil et al. 2020b; Halford et al. 2020; Kirchmaier and Villa-Llera 2020; Office for National Statistics 2020), Australia (Andresen and Hodgkinson 2020; Payne and Morgan 2020a, b), Sweden (Gerell et al. 2020) and Canada (Hodgkinson and Andresen 2020).

**Table 1:** Literature summary of studies

Author(s)	Study area(s)	Crime or incident type	Key findings
Ashby (2020b)	Austin, Baltimore, Boston, Chicago, Dallas, Los Angeles, Louisville, Memphis, Minneapolis, Montgomery County, Nashville, Philadelphia, Phoenix, San Francisco, Tucson, Washington DC	Serious assault (public and residential), burglary (residential and non-residential), theft of vehicles, theft from vehicles	No change in serious assaults (public or residences), some reduction in residential burglary, widespread decline in theft, some variation between cities
Ashby (2020a)	Baltimore, Cincinnati, Los Angeles, New Orleans, Phoenix, San Diego, San Jose, Seattle, Sonoma County, St Petersburg	Calls for service for assault, burglary, dead body, disturbance, domestic violence/family dispute, driving while impaired, drugs, intruder alarm, medical emergency, mental health/concern for safety, missing person, robbery, shooting/shots fired, suspicious person/vehicle, traffic collision, traffic stop, trespassing, vehicle theft	Overall decline in calls for service following stay at home orders, but with some variation. Complex trends (spikes and sudden falls) evident in many cities
Buil-Gil et al. (2020b)	England, Wales, Northern Ireland	Cybercrime (computer virus, malware, spyware, denial of service, hacking, online fraud)	Cyber-dependent crimes increase for individual victims, but largely decrease for organizations. Increase in online shopping fraud for individuals and organizations
Bullinger et al. (2020)	Chicago	Domestic violence crimes and calls for service	Calls for service related to domestic violence increased, especially among blocks spending the most time at home. Decrease in reported domestic crimes and arrests
Campedelli et al. (2020)	Los Angeles	Assault with deadly weapon, battery, burglary, intimate partner assault, robbery, shoplifting, theft, homicides, stolen vehicle	Decline in robbery, shoplifting, theft, battery and overall crime. No change in assault with a deadly weapon, homicides, burglary, intimate partner assault or stolen vehicles
Felson et al. (2020)	Detroit	Burglary (residential and commercial)	Burglary shifted away from residential areas towards mixed-use (including non-residential) areas
Gerell et al. (2020)	Sweden	Outdoors assault, personal robberies, indoors assault, residential burglary, non-residential burglary, pickpocketing, narcotics crime, vandalism	Decline in overall crimes, assaults (indoor and outdoor), burglary (residential and commercial), pickpocketing, theft. No apparent change in narcotics crimes and personal robberies. Increase in vandalism
Halford et al. (2020)	England, Wales	Shoplifting, other theft, domestic abuse, theft from vehicle, assault, burglary (dwelling and non-dwelling), vehicle theft	Decline in shoplifting, theft, domestic abuse, theft from vehicle, assault, burglary (residential and non-residential). Evidence of relationship between crime changes and mobility
Hawdon et al. (2020)	United States	Cybercrime	No major change in cyber-routines or cybervictimization
Hodgkinson et al. (2020)	Vancouver	Burglary (residential and commercial), theft of vehicle, theft from vehicle, theft, violence, mischief	Decrease in total crime, commercial burglary (subsequent increase), theft, theft from vehicles. No clear change in violence, mischief or residential burglary
Mohler et al. (2020)	Los Angeles, Indianapolis	Calls for service for burglary, assault-battery, vehicle theft, domestic violence, vandalism, traffic stops	Some decrease in burglary, robbery. Major decline in traffic stops. Increase in vehicle crimes and domestic violence. Some evidence of relationship with mobility, but many changes marginal
Payne and Morgan (2020a)	Queensland	Common assault, serious assault, sexual offences, domestic violence order breaches	No clear deviation from historical trends
Payne and Morgan (2020b)	Queensland	Property damage, shop theft, other theft, burglary, fraud, motor vehicle theft	Decline in shop theft, other theft and credit card fraud. No change for property damage, burglary or vehicle theft
Piquero et al. (2020)	Dallas	Domestic violence	Short-term spike in domestic violence
Shayegh and Malpede (2020)	San Francisco, Oakland	Theft, homicide, traffic incidents, domestic violence	Decline in overall crime, theft, homicide, traffic incidents. No decline in domestic violence

Findings from this array of research have generally aligned with theoretical expectations from routine activities theory, but there are exceptions and caveats. One conclusion we *can* draw is simply that “crime has changed” in response to restrictions aimed at curbing the spread of the virus (Gerell et al. 2020, p. 2). As Table 1 demonstrates, numerous studies have reported widespread declines in common police-recorded crimes such as a residential burglary, shoplifting, theft and assault. In many cases, these declines hold association with fluctuations in mobility (e.g. Halford et al. 2020) which suggests that lockdowns have disrupted the frequency of convergence between motivated offenders, suitable targets and a lack of guardianship (Stickle and Felson 2020).

In some cases, studies report unexpected or conflicting findings. This appears to be, at least in part, attributable to the short time frames being studied and limitations in the data being used. For instance, Payne and Morgan (2020a) reported no shift in violent crimes recorded in Queensland, Australia, but note that the impact of changes in mobility may not yet have come to fruition during the short study period. Similarly, Hawdon et al. (2020) found that cyber-routines and cyber victimization remained unchanged, but measurements were taken early in the pandemic. Studies have also reported no change (Shayegh and Malpede 2020), short-term spikes (Piquero et al. 2020) and increases (Mohler et al. 2020) in domestic violence. Such issues showcase the challenges of understanding crime during lockdown ‘on the fly’, especially when relying on a single data source, which has typically originated from police-recorded crime databases and covered short time periods.

At the time of writing, the bulk of existing studies cover relatively short periods by necessity, typically addressing only the weeks or months immediately following lockdown. Consequently, we are yet to capture the summer period during which time lockdown guidelines were gradually relaxed. In England and Wales, we are currently lacking examinations into crime which capture both the immediate imposition of lockdown and its gradual withdrawal. With this in mind, the present study analyses six months of police-recorded data from March to August 2020 using fourteen different offence categories. We quantify the extent to which the trajectories observed during the study period deviate from what we might otherwise have expected without the lockdown. We offer an in-depth discussion on findings with reference to theoretical expectations and the data sources used.

### Lockdown and mobility

#### Timeline

The timeline tracing the severity of lockdown guidelines in England and Wales during the study period is fundamental to understanding and interpreting the crime trends observed (Table 2). It is changes in citizen mobility which are expected to dictate the opportunity structures for crime, and in turn, the trends observed in police-recorded data.

**Table 2:** Key lockdown events from March to August 2020

Date	Guidelines
23 March	Government message to citizens is "stay at home". Exceptions for limited purposes, such as shopping for food. Only one outdoor exercise each day. Travel to work is only permitted if absolutely necessary and if the work cannot be done remotely. No mixing with other households
24 March	Government text message sent out via all mobile phone networks to stipulate the "stay at home" message
25 March	Government announces that the police will be authorised in using force to ensure that people align with the lockdown regulations and restrictions on non-essential activities. Some exceptions were stipulated, including for victims of domestic violence
26 March	Lockdown restrictions come into effect through legislation, providing legal force to the lockdown "stay at home" guidelines
3 April	Figures from the Cabinet Office demonstrate substantial drops in transport usage including motor vehicles, national rails and buses. Cycling has increased
16 April	Nationwide lockdown measures are extended for a further three weeks
10 May	The government's official message of "stay at home" changes to "stay alert". The Prime Minister announces that initial steps will be taken to relax lockdown measures. Some professions are encouraged to return to work (e.g. those in construction) but avoid public transport
13 May	Restrictions begin lifting, including the re-opening of garden centres, sports fields and recycling centres
18 May	Rail networks begin to increase services
1 June	The "rule of six" is implemented, which stipulates that people from more than one household can meet but only outdoors. Further relaxation of restrictions, with outdoor sport facilities and outdoor markets re-opening
13 June	Government permits "support bubbles" which permit two households to meet but only in cases where one household is a single adult or single adult with a dependent child
15 June	Retail shops are permitted to re-open but hospitality venues such as bars, pubs and restaurants remain closed
25 June	Short heatwave prompts major domestic transport spike, especially in coastal areas
29 June	Local lockdown measures introduced for the city of Leicester
4 July	Major relaxation of restrictions (excluding Leicester) permitting the re-opening of venues including pubs, cafes, bars, theatres and places of worship, with some restriction. Groups including up to two households can meet in public or private spaces, indoors or outdoors
17 July	Rules relaxed on public transport, permitting non-essential usage
1 August	Shielding guidelines for the most vulnerable cease in England, permitting 2 million vulnerable people to leave their home and return to work
29 August	First football match with spectators since lockdown began is held

On 16 March, the UK government recommended the cessation of all non-essential travel and contact between persons, and on 23 March announced the first ‘stay at home’ order. Measures were designed and expected to be followed overnight. The official message became “Stay at Home, Protect the National Health Service, Save Lives”. In the days that followed, these restrictions become legally enforceable, and the police were authorized to use force to ensure that people were following the rules. April was the first full month of the nationwide lockdown. As restrictions slowly began having their intended impact, some were slowly repealed or relaxed. The first of June marked an important date in this respect, permitting people from more than one household to meet outdoors up to a maximum of six people. This was soon followed by the introduction of “support bubbles” which allowed two households to meet indoors under specific circumstances, such as when one household comprised a single adult with a dependent child. On 4 July, venues such as pubs, cafes and places of worship re-opened, although limits remained on the number of households permitted to meet. By the beginning of August, “shielding” guidelines for clinically vulnerable people were lifted.

#### Mobility

Google Mobility Reports provide aggregated, daily, anonymized information on ambient population movements at a sub-regional level in the United Kingdom. The data underlying these reports was made openly available early in the COVID-19 pandemic in an effort to help the public, government and researchers understand how “stay at home” (and equivalent) policies were impacting on mobility. The information has quickly become a useful source of information to study the relationship between crime and mobility during the pandemic (Halford et al. 2020; Mohler et al. 2020). Here, we make use of the raw data from these reports to visualize how mobility changed in response to the nationwide lockdown measures. This provides a key aspect of the context for our subsequent analysis and findings using police-recorded crime data.

**Fig. 1:**
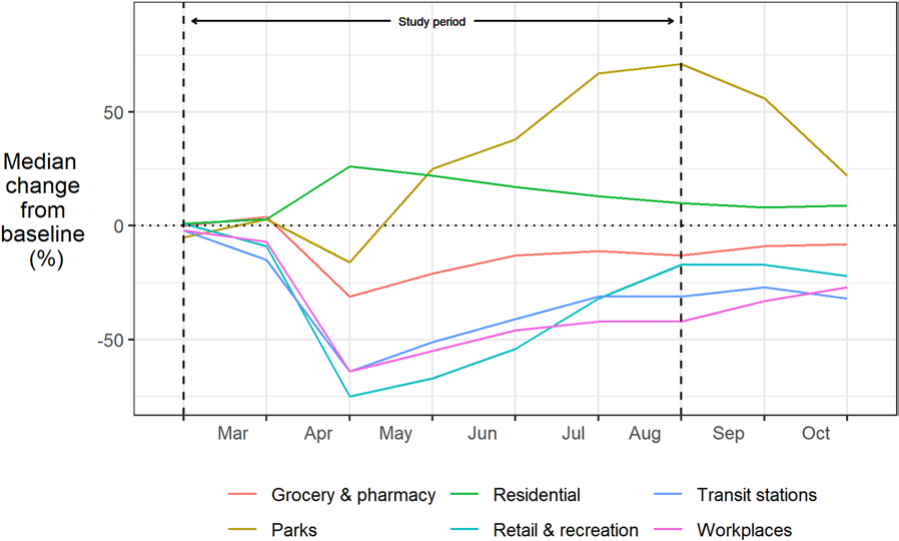
Median percentage change from baseline mobility for subregions of England and Wales (excluding Greater Manchester) in 2020. Raw data obtained from Google Mobility Reports

The data provides a measure of the percentage change in mobility compared to a baseline figure considered to be ‘typical’.^2^ A positive percentage indicates higher mobility compared to the baseline, and vice versa. From these raw figures we can calculate the median percentage change in mobility across sub-regions in England and Wales by month (see Fig. 1). To match the study region and corresponding police-recorded crime data, we excluded Greater Manchester, as detailed in the following section.

Even by the end of March, after only days of official “stay at home” restrictions, the ambient population in residential areas was higher than the ‘typical’ baseline, as people began adhering to the new rules. With people forced to spend time at home, mobility dropped in workplaces, transit stations and in retail and recreational areas. The scale of this change would become even more evident in April, the first full month of lockdown. With limits on outdoor exercise, mobility also decreased in parks during April.

Since then, although we have witnessed a gradual convergence back towards the baseline for most mobility types, people’s routine activities remain far from typical. Even by August, by which time pubs and restaurants were open, and the government was encouraging many employees to return to ‘on site’ work, mobility in residential areas remained unusually high. Similarly, despite an initial turnaround, mobility in shopping, retail and workplace areas, along with transit stations, had leveled-off below the baseline. Noting a potential seasonal effect, the usage of parks increased dramatically compared to the baseline once limits on using exercise and outdoor socializing were relaxed, peaking at the end of the study period. Although the longevity of these changes may not be known for some time, we can be certain that the lockdown induced an unprecedented shift in people’s mobility and routine activities.

**Fig. 2:**
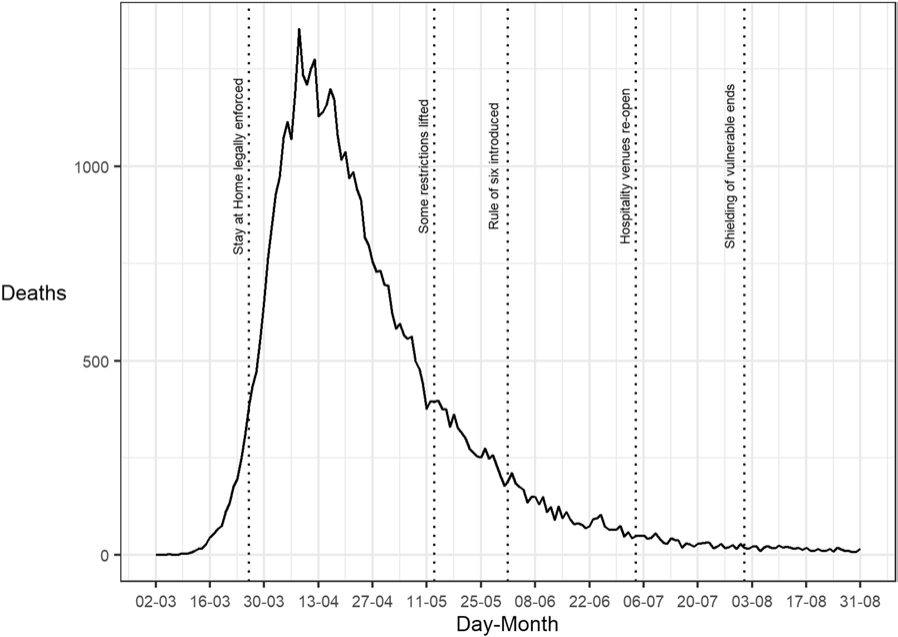
Deaths in England and Wales for which COVID-19 was mentioned on the death certificate during the study period. Labelled with key lockdown dates. Raw data sourced from Office for National Statistics

The impact of these mobility patterns on the spread of the virus is demonstrable (see Fig. 2). Daily deaths attributable to COVID-19 soared during March, but quickly began to decline during April, the first full month of lockdown. Deaths continued to decline throughout spring and summer. The trends observed reflect widespread compliance with the regulations in England and Wales during the study period. Given that open police records, outlined in the next section, are aggregated data by month, it is noteworthy that many (but not all) major changes in lockdown guidelines occurred on or near to the beginning of a month.

It is clear, then, that the nationwide lockdown enforced throughout March and August produced dramatic changes in people’s routine activities. Mobility was severely curtailed on a national level. In turn, the spread of the virus slowed and mortality rates began to decline. Drawing upon opportunity theories of crime, and following Halford et al.’s work (2020) that more directly explores the crime-mobility link, we expect that the stark changes in people’s mobility observed in England and Wales between March and August 2020 were largely responsible for the similarly stark fluctuations in crime that we identify in what follows.

## Data and method

### Crime data

To examine the extent to which crime changed during the imposition and relaxation of nationwide “stay at home” measures, we make use of open data on police-recorded crime and anti-social behavior. Data was compiled from 42 out of 43 police forces across England and Wales. Greater Manchester Police did not publicly release sufficient amounts of data due to issues switching to a new computer system in 2019, and thus were excluded from analysis. Data is released on a monthly basis via an open data portal for each force (https://data.police.uk/) and archived from previous years to permit analysis of historical trends.

To assess the extent to which trends in crime and anti-social behavior observed during the pandemic differed from what we would otherwise have expected, data was collated from March 2015 to August 2020. The period March 2015 to February 2020 was used to model the ‘expected’ trend, as detailed in the next section. The data covering March to August 2020 covers the first 6-months of the nationwide lockdown.

Open police-recorded crime data contains individual records of offences categorized according to thirteen crime types deemed to be ‘notifiable offences’ according to the National Crime Recording Standards. These crime types are defined by aggregating across sub-classes. For instance, ‘violence and sexual offences’ includes homicide, rape and the use of firearms to resist arrest, amongst others. Anti-social behavior (ASB) is not considered to be a notifiable offence and is usually reported separately from ‘crimes’ in national statistics. ASB includes less serious offences such as nuisance behavior. A summary of these categories is detailed in Table 3.

**Table 3:** Offence categories in open police-recorded crime data. Source: www.police.uk

Crime category	Description
Anti-social behaviour	Personal, environmental and nuisance anti-social behaviour
Bicycle theft	Taking without consent or theft of a pedal cycle
Burglary	Person enters a house or other building with the intention of stealing
Criminal damage and arson	Damage to buildings and vehicles and deliberate damage by fire
Drugs	Offences related to possession, supply and production
Other crime	Forgery, perjury and other miscellaneous crime
Other theft	Theft by an employee, blackmail and making off without payment
Possession of weapons	Possession of a weapon, such as a firearm or knife
Public order	Offences which cause fear, alarm or distress
Robbery	Offences where a person uses force or threat of force to steal
Shoplifting	Theft from shops or stalls
Theft from the person	Crimes that involve theft directly from the victim (including handbag, wallet, cash, mobile phones) but without the use or threat of physical force
Vehicle crime	Theft from or of a vehicle or interference with a vehicle
Violence and sexual offences	Offences against the person such as common assaults, Grievous Bodily Harm and sexual offences

Individual crime and ASB records were aggregated by type and by month. Counts were adjusted by the resident population using mid-year estimates, excluding the population of Greater Manchester to reflect the lack of police data for the region. We assumed that population growth is uniform between months, and that population growth in Greater Manchester is the same as the rest of the country. The final dataset for analysis consisted of monthly crime rates (by 10,000 resident population) for the thirteen crime types and ASB (see Table 3) between March 2015 and August 2020 in England and Wales.

### Crime model

As noted, the principal aim of this study is to determine the extent to which the COVID-19 pandemic impacted on crime in England and Wales. The expectation is that dramatic changes in people’s mobility and social interactions, brought about by nationwide restrictions to curb the spread of the virus, will have brought about similarly dramatic changes in crime. We cannot determine this by studying the lockdown period in isolation. To understand the extent of the change, we need to construct an expectation of what the crime rate might have been if the pandemic had not occurred. That is, construct counterfactual crime rate estimates for the period of the pandemic based upon the premise that the pandemic did not happen. To this end, we use Autoregressive Integrated Moving Average (ARIMA) models to construct a ‘short term forecast’ using data prior to the pandemic.

Police-recorded crime trends can vary considerably over long periods of time. They can be subject to short-term fluctuation and seasonal trends (McDowall et al. 2012). Police crime statistics can also fluctuate due to recording practices and the willingness of victims to report crime. Forecasting models will ideally account for this kind of variation in order to make meaningful comparisons. As with any forecasting models, due consideration of previous trends and values is paramount for accurate forecasts, and therefore the forecasts that are built have implicit assumptions regarding the continuation of previous patterns embedded within them. Time series models, including the ARIMA models that we deploy here, are capable of accounting for this historic variation, but will also be subject to the implicit assumptions of trend continuance.

The forecasting of crime rates tends to fall into the domain of predictive policing (Kounadi et al. 2020) which forecasts both spatial and the temporal dimensions. As this study was only concerned with one geographical area, England and Wales, we focus on temporal forecasts. ARIMA models account for the trends and seasonality that typically affect crime rate forecasts, and as such, have an established pedigree in criminological research (Chamlin 1988; Chamlin and Cochran 1998; Chen et al. 2008; Leitner et al. 2011; Moffatt et al. 2012).

Crime and ASB rates between March 2015 and February 2020 were used as the baseline to generate the expected rates. In general, the data for time series models is decomposed to allow the calculation of estimates for the seasonal component and the trend. Once this is completed, the de-trended and de-seasoned residuals are analyzed to determine the coefficients of the ARIMA model. The model is then reconstructed from the ARIMA coefficients, the seasonal components and the trend components. This model is then used to make predictions, with the unexplained variance reflected in the confidence intervals surrounding the point estimates. Here, the *forecast* package (Hyndman and Khandakar 2008; Hyndman et al. 2020) in R (R Core Team 2013) was used as an end-to-end process to conduct the steps in the time series modelling. The *forecast* package deploys an automated, step-wise method for identifying the best model fit by minimizing the Akaike Information Criterion (AIC).

Allowing for seasonal variation, the final model provided point estimates of crime rates between March to August 2020, along with 95% confidence intervals to reflect a reasonable level of uncertainty in the estimates. These expected trends could then be compared to the observed rates. In a scenario in which the observed rates overlap with the confidence intervals around the estimates, there would be no evidence to suggest that crime has deviated from what we would have expected in the absence of the COVID-19 pandemic and resulting lockdown. For ease of interpretation, we also visually report the percentage difference between the point estimates and observed rates. Code for the analysis can be found in the Additional file 1.

## Results

**Fig. 3:**
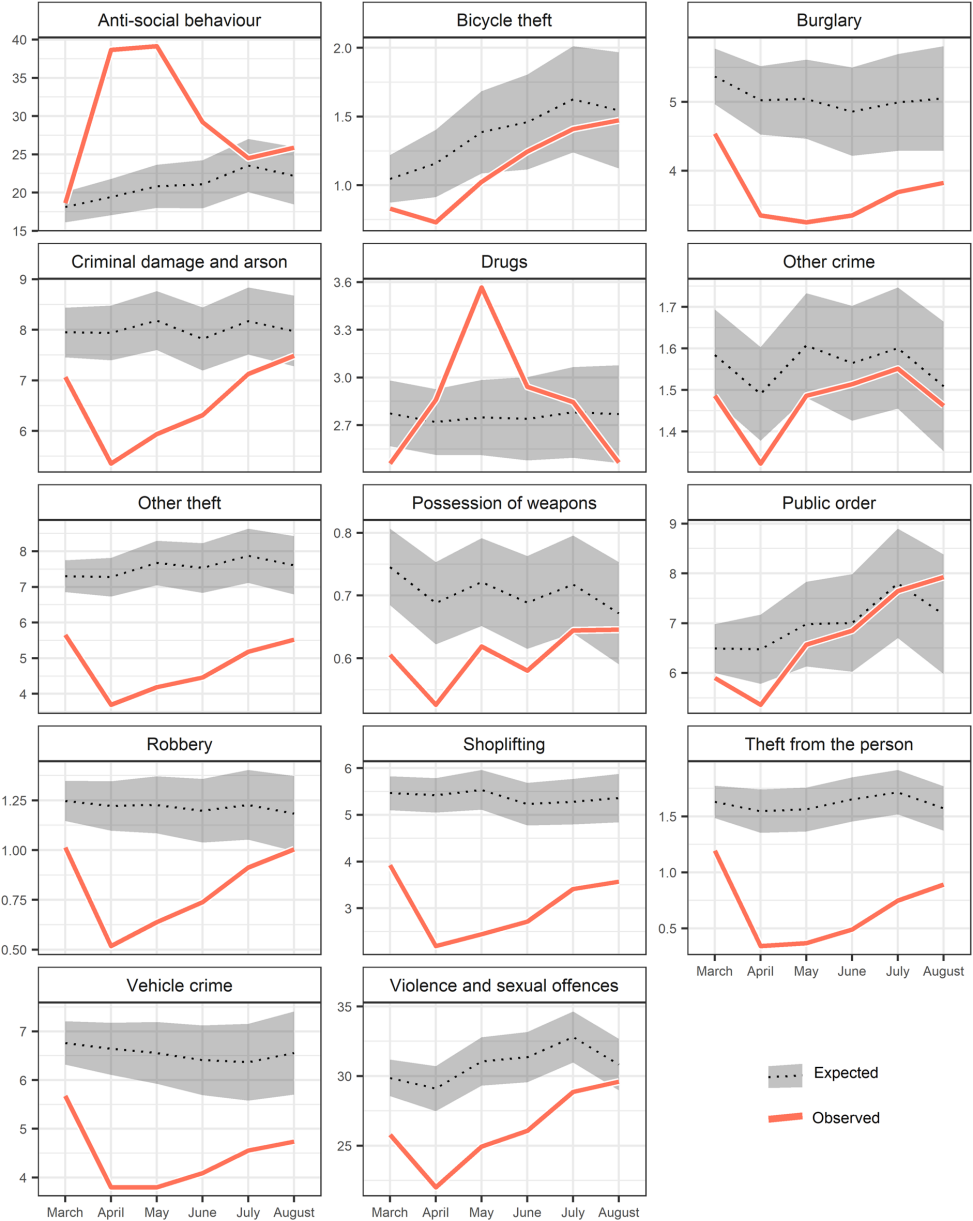
Observed crime and anti-social behavior rates per 10,000 population (in red) between March and August 2020 compared to expected trend (in dotted black) using 95% confidence intervals (in grey)

**Fig. 4:**
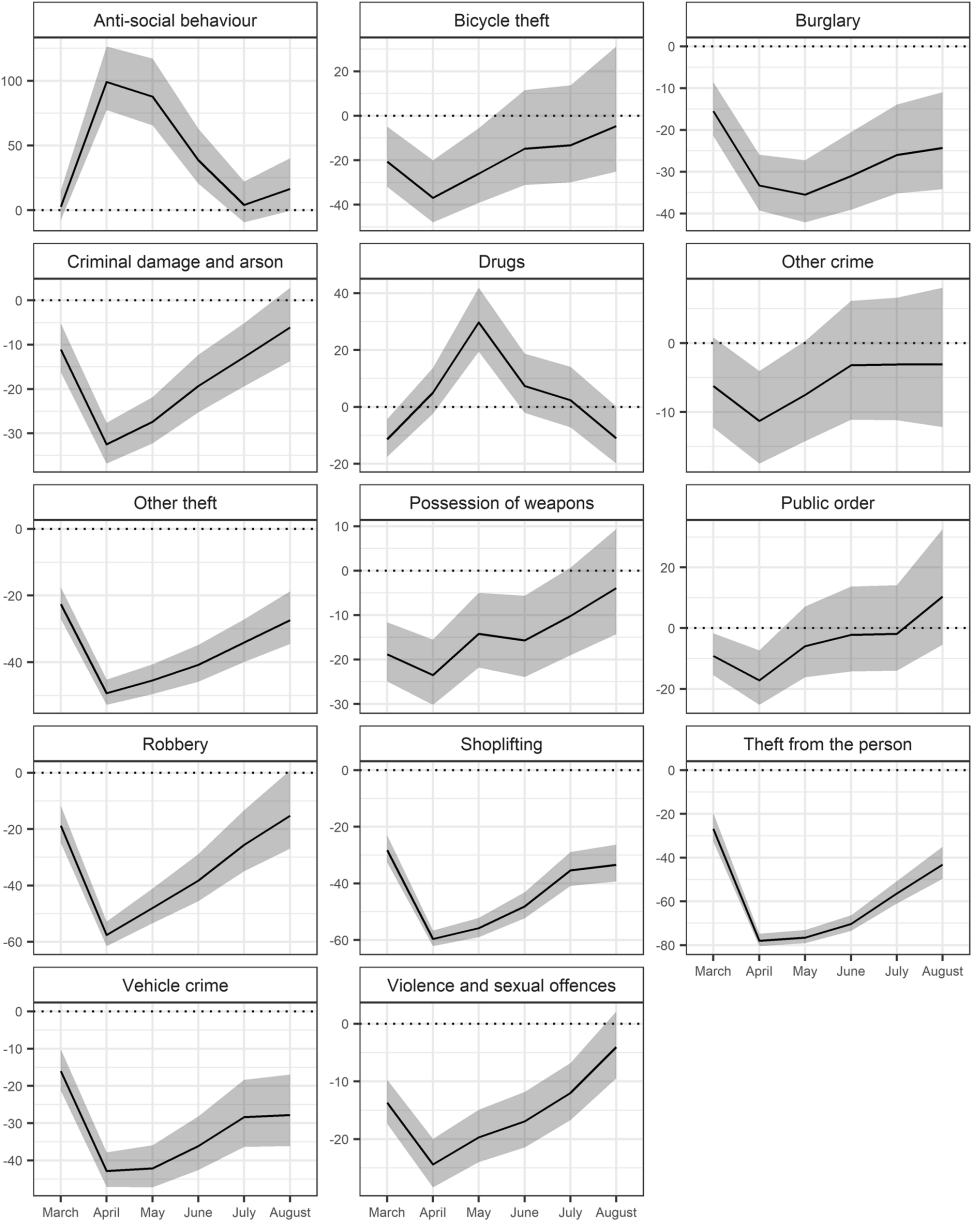
Percentage difference (in black) between observed crime and anti-social behavior rates for March to August 2020 using 95% confidence intervals (in grey) relative to expected trend. Reference line for no difference is 0% (in dotted black)

Findings from the ARIMA analysis are reported using two different but complimentary visualizations. First, the crime rates *observed* during lockdown are plotted against point estimates of the crime rates we would have *expected* during the same period in the absence of a pandemic (Fig. 3). The confidence intervals either side of the expected rates convey the degree of uncertainty around these estimates. Second, we plot the *percentage difference* between what was observed during the lockdown and the point estimate of what was expected, along with the respective confidence intervals (Fig. 4).

ASB and drug crimes were the two offence types which experienced *increases* during the lockdown period relative to what would otherwise have been expected. By the end of March, following one week of lockdown measures, rates of ASB were within the range we would have expected. But, following the first full month of restrictions, ASB skyrocketed. The volume of ASB observed during April was 100% higher than what we expected based on typical seasonal variations and long-term trends. This sustained itself into May, followed by a sharp decline as lockdown restrictions were eased. By July, ASB had returned to usual levels, although there is evidence of a revival in August. It is noteworthy that some police forces were reported to have recorded breaches of lockdown rules during the pandemic using ASB.

Drug crimes are the only notifiable offence to experience an increase during April, having begun from an exceptionally low starting point in March. This surge continued into May, by which point rates were 30% higher than expected. In the months following this spike, rates declined over consecutive time periods. By August, the data suggests that the volume of drug offences being recorded by police might have been even *lower* than expected. Here, we would emphasize that the trends observed for drug offences likely reflect policing activity and the ease of arrest, rather than a shift in criminal behavior. This point is returned to in the discussion section.

Types of theft and robbery experienced remarkably similar trends. The impact of the lockdown on robbery was immediate and dramatic. By April, robbery was nearly 60% below what we would have expected. That said, it demonstrated an ability to ‘bounce back’. Observed robbery rates reached within the range to be expected for August. This is likely to mirror mobility changes and reflect a partial return to previous forms of convergence of offenders, targets and guardianship.

Rates of theft from the person saw the most significant decline. In April, rates were nearly 80% lower than what we would have expected without a pandemic. Subsequently, the return towards expected levels was gradual. Rates remained low into May, then crept back up, but by August, remained at an unusually low level. Other forms of theft, which includes key categories such as making off without payment, were already over 20% below expected in March, dropping further to around 50% in April. Since then, we have witnessed the beginning of a return towards expected levels, but the slope has been shallow. By August, the scale of other theft remained considerably lower than expected. Shoplifting experienced a similarly stark decline, bottoming-out at 60% below expected in April. That said, even amidst the relaxation of rules governing the closure of commercial outlets, the resurgence towards expected levels was slow. In August, the bounce back slowed, and shoplifting remained around 30% lower than we would have expected at the end of the study period.

We note that crimes that often occur in residential areas, such as bicycle theft, burglary, vehicle crime, and criminal damage and arson, demonstrated similarly stark patterns. Even in March, burglary was around 15% down on what we might have otherwise expected. By April and May, burglary rates were a third below the levels expected in the absence of a pandemic, but increased from June. Even at the end of the study period, when lockdown restrictions had been eased, burglary remained significantly below the expected level. In fact, the initial resurgence appeared to have tailed off into August, suggesting that burglary may not return to typical levels for some time. This may reflect a more permanent shift of day-time populations to residential areas as many people continued to work from home, acting as capable guardians, discussed further later. The trend in vehicle crime was similar to that of burglary and may well reflect similar changes to lifestyles.

Rates of bicycle theft were already below normal at the end of March, declining further into April. Here, at its lowest level, bicycle theft was nearly 40% lower than what we would have expected. Rates of bicycle theft subsequently increased. By June, the observed rates were overlapping with the range of uncertainty in the expected estimates. We conjecture that widely reported increased cycling during the pandemic (BBC 2020a, b) increased the ease and attractiveness of bicycle theft which meant that, in turn, its rate increased more quickly than other types of crime.

Criminal damage and arson fell marginally during March, and declined dramatically during April. It demonstrated a steady return towards expected levels through August, by which time rates were within the range we would have expected without a pandemic. The possession of weapons, which includes firearms and knives, was around a quarter below expected levels in April. It then increased, albeit with a June blip, returning to expected levels by August.

Public order was 20% below expected by April, which is a less pronounced decline than many crime types. By May, the rate of public order recorded had returned close to the levels expected in the absence of a pandemic—an increase which continued into August. Public order offences include offences such as alcohol-related disorder, which may interact with lockdown breaches and similar offences, but further research into the nature of change in public order offences is needed. The Crown Prosecution Service reported that public order offences were the third most likely category of offences to be categorized as ‘coronavirus-related’ in the first six months of the pandemic following ‘coronavirus offences’ and assaults on emergency workers (Crown Prosecution Service 2021).

Other crimes, representing a diverse group of offences (see Table 2), only experienced unprecedented levels in April. Although the point estimate for the expected rate remained higher than the observed rate throughout the study period, the confidence intervals overlap between May and August, suggesting that lockdown had a very short-term impact on these crime types.

Violence and sexual offences represent the most frequently occurring notifiable offence type in open police records. In April, the crime rate dropped sharply, to 24% below expected. As with many other crime types, this initial fall was followed by an increase back to expected levels over the following months. By August, the observed crime rate had bounced-back to within a range we might have otherwise expected without the pandemic and restrictions on mobility. Here, it is worth noting domestic-specific instances of violence cannot be identified.

In the Appendix we also report a descriptive comparison between the crimes rates observed during lockdown, and those crime rates observed during the same period in 2019. This provides a sensitivity check on the estimates generated from the ARIMA analysis. The descriptive comparison broadly confirms the main findings from this study. The only crime type with notable discrepancies is violence and sexual offences, for which the trend is identical but the difference between observed and expected is reduced when using 2019 as the baseline. For instance, violence and sexual offences were 15% lower in April 2020 compared to April 2019, but ~25% lower compared to the ‘expected’ point estimate. The trajectory of the subsequent resurgence was comparable, but by August, violence and sexual offences in 2020 were 8% *higher* than the same month in 2019. We suspect that the difference for violence and sexual offences reflects the continuance of the upward trend in the ARIMA model that is not accounted for in the descriptive year-to-year comparison.

## Discussion

The findings presented here strongly suggest that crime in England and Wales changed considerably in response to the COVID-19 pandemic. We suspect that these changes occurred as a direct response to dramatic changes in people’s lifestyles and mobility. This study included both the introduction and subsequent relaxation of lockdown guidelines, and thus captured the resurgence of many crime types following initial declines. Findings highlight a number of points for discussion both in relation to theory as well as the limitations of using police data to study this ‘natural experiment’.

Generally speaking, findings align with expectations from opportunity-based perspectives on crime. It was predicted that, for many crime types, the enforcement of “stay and home” measures would change lifestyles and mobility, thereby unsettling the convergence of motivated offenders, suitable targets and capable guardians. As noted, existing studies using short time periods tended to support this hypothesis, with the introduction of restrictions resulting in declines across multiple different crime types. Here, we note similarly, but also demonstrate both directions of the relationship, having showcased the beginning of a resurgence back to expected levels of crime as restrictions on mobility were slowly eased. This represents a key component of the dose–response causal relationship between mobility and crime.

In England and Wales, twelve out of the fourteen offence categories in open police records experienced a sharp decline in the first full month of lockdown, falling below what we would have otherwise expected. For some of these crime types, the impact of the lockdown may well stretch far beyond the conclusion of the pandemic. The decline in crimes such as burglary and vehicle crime likely reflect a swell of daytime populations in residential areas, increasing capable guardians and ‘eyes on the street’. However, their resurgence has been slow. The canon of theory and evidence relating to crime displacement suggests that longer-term adaptation to other crime types by offenders will be the exception rather than the norm (Guerette and Bowers 2009). This makes us optimistic that the prospect of long-term shifts towards home-working may well keep these largely residential crimes permanently below historical levels.

The closure of non-essential shops, and subsequent decline in mobility in and around retail areas, clearly reduced the number of target enclosures available to shoplifters during lockdown. The return of shoplifting from May onwards was slow relative to some crime types, and even tailed-off towards the end of summer. Even once open, shops have tended to enforce restrictions on the number of people entering to facilitate social distancing, removing the anonymity of crowds, and making potential shoplifters easier to spot by security personnel and witnesses. Moreover, the longer-term continuity of high street shopping is no longer guaranteed. Lockdown measures have represented a particularly fortuitous moment for online retailers. The prospect of a permanent decline in daytime city center populations, as people continue to work from home, may act as a catalyst for the decline of the high street, and in turn, opportunities for shoplifting.

The initial decline in bicycle theft is consistent with lockdown rules which limited outdoor activity (and other non-essential activities) to only once per day. In April, people were unable to leave their homes frequently, and few places were open which would require leaving a bicycle unattended (locked or otherwise), drastically reducing the number of suitable targets. Cycling increased, as noted above, and bicycle theft returned to the expected range as restrictions on outdoor activity were relaxed. However, it remains unknown to what extent the surge in cycling, increasing potential target availability and stimulating the secondhand market on which stolen bicycles can be sold, will continue in a post-pandemic era (Hong et al. 2020).

While open police-recorded crime has facilitated the analysis presented here, as with numerous existing studies examining crime during the COVID-19 pandemic, its usage merits a discussion in its own right. This is particularly pertinent given the tendency of crime science research to study the pandemic as a ‘natural experiment’. Firstly, it remains unknown to what extent existing issues in police-recorded crime data, such as underreporting and sensitivity to police training and activity (Buil-Gil et al. 2020a; Schnebly 2008) have been remedied or exacerbated by the pandemic. This adds a confounding factor to the experiment, and in such a scenario, it would be problematic to attribute changes in police-recorded crime solely to changes in the opportunity structure of crime during the pandemic.

By way of an example, drug crimes represent the only notifiable offence to have *increased* above and beyond expected levels during the nationwide lockdown in England and Wales. Yet, we suspect that the increase is unlikely to reflect a meaningful shift in criminal behavior. Firstly, reports have suggested that drug activity has simply become more visible on empty streets, making offenders easier to spot and arrest (Langton 2020). Secondly, trends in police-recorded drug offences, such as dealing, can be proxy measures for the proactivity of the police (Ariel and Bland 2019). The sudden fall in other crime-based police demand, brought about by lockdown restrictions and the ensuing limits on crime opportunities, will have occurred in concert with other changes, such as the cancellation of major sporting events which would otherwise have drained substantial police resource. As such, the increase in police-recorded drug offences likely represents a salient example of proactive policing in the absence of typical police demand.

Similarly, the National Police Chief’s Council (NPCC) has largely credited the increase in ASB to police enforcing lockdown guidelines. In which case, the gap between what we *expected* and what was *observed* would predominantly reflect the extent to which people adhered to lockdown guidelines, and/or the amount of surplus resource police had to tackle lockdown breaches. That said, the proportion of excess ASB that we can attribute to lockdown breaches, and the extent to which this might vary between police forces, remains unknown.

## Conclusion

This study has provided an initial ‘look back’ on police-recorded crime and anti-social behavior (ASB) during the first six months following nationwide lockdown in England and Wales. We used Autoregressive Integrated Moving Average (ARIMA) models to estimate the amount of crime we would have expected *without* the COVID-19 pandemic and ensuing restrictions on mobility. These estimates were then compared to the crime rates actually observed during lockdown. We found that twelve out of fourteen offence categories experienced significant declines upon the introduction of lockdown guidelines, followed by a resurgence as restrictions were relaxed. That said, the severity of this ‘bounce back’ varied between crime types. Evidence suggests that residential crimes, in particular, may not return to normality for some time, if at all. Other common crimes, such as robbery and violence (including sexual offences) experienced a rapid return to normality. Findings appear to be consistent with expectations from the opportunity structure of crime. That said, dramatic *increases* in drug crimes and ASB may not be directly attributable to meaningful changes in criminal behavior. This demonstrates the nuances in using police-recorded crime data to study the lockdown as a natural experiment.

It is possible to offer some informed speculation about what the future holds. The mobility theory of crime in the pandemic with which this study is consistent suggests that further iterations of national and local lockdown will cause further national and local declines in most recorded crimes. The magnitude of the crime effect is likely to match the severity of the lockdown restrictions and their impact on mobility (see Dixon and Farrell 2020b for a preliminary glimpse at the less pronounced effect of the less restrictive second lockdown in November). Further, a post-pandemic era seems unlikely to see crime return to the levels expected absent a pandemic. With mobility a key determinant of crime opportunity rates, if ‘work at home’, online shopping and other lifestyle changes continue at higher rates, we might expect commensurate effects upon crime in the longer-term.

### Supplementary Information

**Additional file 1.** Link to the GitHub repository containing code for replicating analysis.

## Figures and Tables

**Fig. 5:**
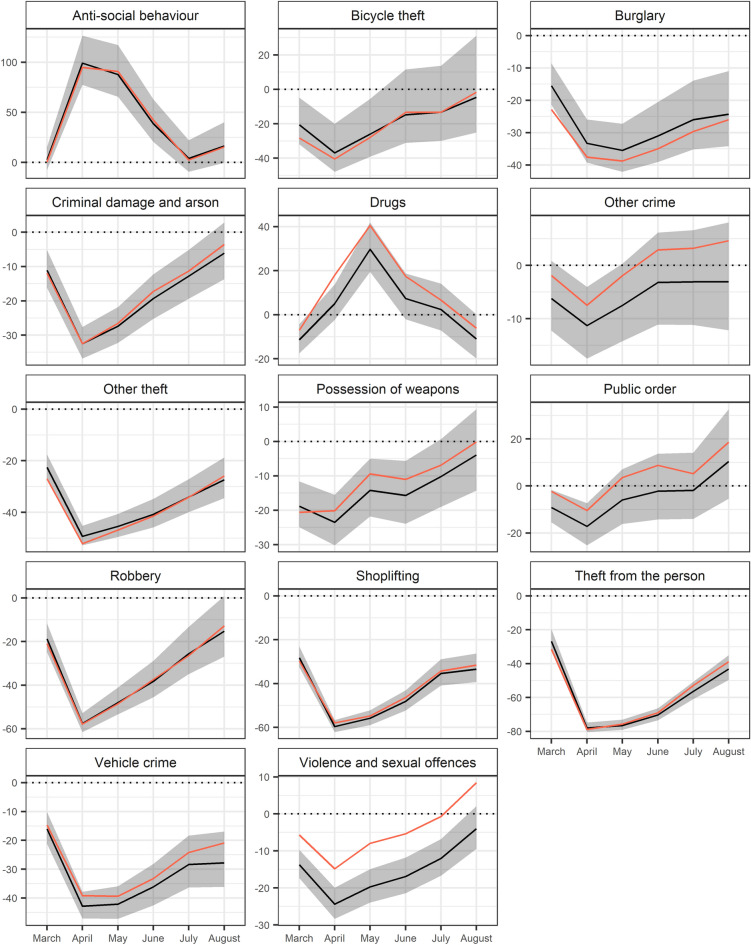
Percentage difference in crime and anti-social behavior observed in 2020 compared to 2019 (in red) and based on previous years (in black) using 95% confidence intervals (in grey).

## Data Availability

The data are publicly available and retrievable from the open online data portal for England, Wales and Northern Ireland. A link to the code used for analyses has been supplied in the supplementary materials.

## Appendix

See Fig. 5.

## Footnotes

1 Available at www.covid19-crime.com.

2 This ‘typical’ baseline is calculated based on data between January and February 2020, and therefore does not account for seasonality. As such, deviations from the baseline may in part be driven by typical seasonal fluctuation, such as the increased usage of parks in the summer. Unfortunately, the lack of open mobility data from Google in previous years makes it impossible to compute a seasonally-adjusted baseline.
